# Pathological Diagnosis of Adult Craniopharyngioma on MR Images: An Automated End-to-End Approach Based on Deep Neural Networks Requiring No Manual Segmentation

**DOI:** 10.3390/jcm11247481

**Published:** 2022-12-16

**Authors:** Yuen Teng, Xiaoping Ran, Boran Chen, Chaoyue Chen, Jianguo Xu

**Affiliations:** 1 Department of Neurosurgery, West China Hospital of Sichuan University, Chengdu 610041, China; 2 Department of Neurosurgery, Ziyang People’s Hospital, Ziyang 641300, China

**Keywords:** craniopharyngioma, MRI, convolutional neural network, computer-aided diagnosis, pathological diagnosis

## Abstract

Purpose: The goal of this study was to develop end-to-end convolutional neural network (CNN) models that can noninvasively discriminate papillary craniopharyngioma (PCP) from adamantinomatous craniopharyngioma (ACP) on MR images requiring no manual segmentation. Materials and methods: A total of 97 patients diagnosed with ACP or PCP were included. Pretreatment contrast-enhanced T1-weighted images were collected and used as the input of the CNNs. Six models were established based on six networks, including VGG16, ResNet18, ResNet50, ResNet101, DenseNet121, and DenseNet169. The area under the receiver operating characteristic curve (AUC), accuracy, sensitivity, and specificity were used to assess the performances of these deep neural networks. A five-fold cross-validation was applied to evaluate the performances of the models. Results: The six networks yielded feasible performances, with area under the receiver operating characteristic curves (AUCs) of at least 0.78 for classification. The model based on Resnet50 achieved the highest AUC of 0.838 ± 0.062, with an accuracy of 0.757 ± 0.052, a sensitivity of 0.608 ± 0.198, and a specificity of 0.845 ± 0.034, respectively. Moreover, the results also indicated that the CNN method had a competitive performance compared to the radiomics-based method, which required manual segmentation for feature extraction and further feature selection. Conclusions: MRI-based deep neural networks can noninvasively differentiate ACP from PCP to facilitate the personalized assessment of craniopharyngiomas.

## 1. Introduction

Craniopharyngioma is a type of rare benign epithelial tumor located along the craniopharyngeal duct, accounting for approximately 1.2–4.6% of all intracranial tumors, with an annual incidence rate of 0.5–2.5 cases per 1 million in the population [1]. According to the latest World Health Organization (WHO) classification of central nervous system (CNS) tumors, craniopharyngioma molecularly consists of two distinct subtypes: adamantinomatous craniopharyngioma (ACP) and papillary craniopharyngioma (PCP) [2]. The clinical manifestations of ACP and PCP can be different; ACP has a bimodal age distribution, with peak incidences between the ages of 5 and 15 as well as 45 and 60, while PCP occurs almost exclusively in adults, peaking between the ages of 40 and 55 [3]. Surgical resection is still the first-line management in the vast majority of cases, but there is a high rate of postsurgical comorbidities that significantly impair daily functions [4]. In addition, patients with ACPs may suffer from higher recurrence rates and poor prognoses compared to PCP, mainly due to their infiltrative nature and low complete-resection rate [5,6].

The recent development of genomics allows clinicians to explore a more individualized method for craniopharyngioma treatment and prognostic prediction. It has been identified that the CTNNB1 mutation is mostly associated with ACP, while PCPs frequently harbor the somatic BRAF-V600E mutation [7,8,9]. A targeted therapy harboring BRAF-V600E is therefore viable for PCP treatment, and it has been reported to be feasible in several individual cases [10,11,12,13]. Moreover, PCP tends to present a higher overall expression of PD-L1 than ACP, which seems to be associated with the BRAF mutation, and may have a favorable response to anti-PD-L1 therapy [14]. Therefore, a combination of targeting PD-L1 and a BRAF inhibitor is an attractive potential therapy for PCP patients [6,15]. Overall, given these progresses in individualized treatment and the distinction in patient prognosis, molecular diagnosis before treatment is rather clinically relevant and should be explored. Lacking specific clinical symptom or blood biomarkers, magnetic resonance imaging (MRI) is the standard as well as the most important preoperative assessment of craniopharyngioma. On T1-weighted images (T1WI), a lobulated, hyperintense, cystic appearance is commonly observed in ACP, while a spherical, hypointense, solid appearance with cysts is observed in PCP [16]. However, the differentiation of ACP and PCP based on above image characteristics is subjective and inadequate, especially for adult patients [16,17]. Nowadays, a series of artificial intelligence (AI) models have been built to assist radiologists and have shown gratifying results in lesion detection, segmentation, diagnosis, and genetic prediction [18,19,20,21]. Previously, several machine learning models built with selected radiomics features were used to discriminate ACP from PCP and to estimate the BRAF and CTNNB1 mutation statuses in craniopharyngioma [22,23,24] although the results were laborious, as they required radiomics features extracted from manual segmentation and a selection of appropriate features [25,26]. Convolutional neural networks (CNNs), a class of deep learning technology, allow for the automatic recognition of important features for detection or classification through multiple layers of representations in raw data [27,28]. Thus, an automated end-to-end approach based on deep learning technology requiring no human involvement should be investigated. In this study, we retrospectively analyzed the clinical and MRI characteristics of ACP and PCP in adult patients. Moreover, we developed a series of CNN models to automatically discriminate ACP from PCP using routine MRIs and tested their performance with classical radiomics methods.

## 2. Materials and Methods

### 2.1. Patient Enrollment

**Figure 1:**
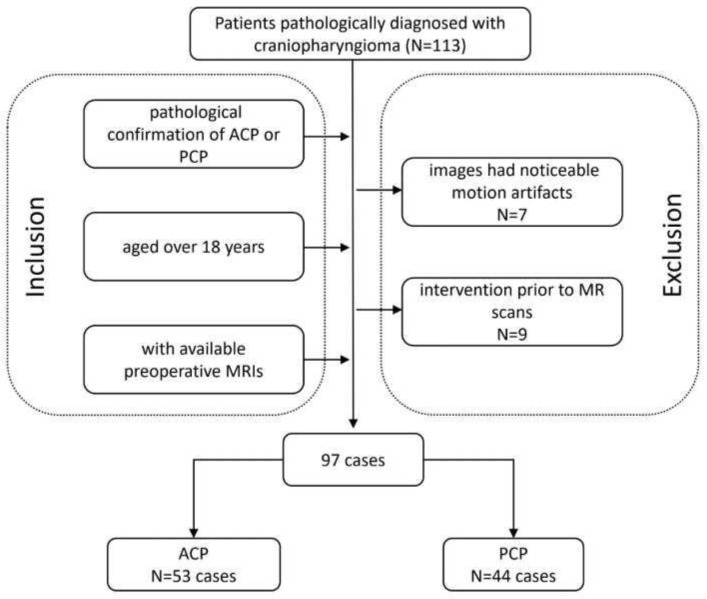
Flowchart of the involved population. ACP, adamantinomatous craniopharyngioma; PCP, papillary craniopharyngioma.

This was a single-center retrospective study performed in the radiology department of West China Hospital. We reviewed the electronic records of patients who underwent tumor resection and were diagnosed with craniopharyngioma between June 2015 and December 2020. The inclusion criteria were as follows: (a) patients with a pathological confirmation of ACP or PCP; (b) patients aged over 18 years at the time of diagnosis; and (c) patients with available preoperative MRIs. The exclusion criteria were (a) images that had noticeable motion artifacts and (b) therapeutic or diagnostic interventions prior to MR scans, such as radiotherapy and biopsy. The clinical characteristics of the patients and the radiological characteristics of the tumors were also evaluated and collected. The working flow chart of this study is shown in Figure 1. This retrospective study was approved by the institutional review board of West China Hospital, Sichuan University, and the informed consent was waived (2021-S-851).

### 2.2. MRI Protocol

**Figure 2:**
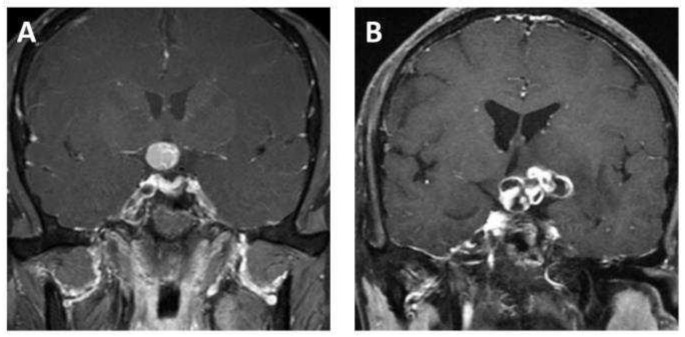
Representative contrast-enhanced T1-weighted MR images of (**A**) adamantinomatous craniopharyngioma and (**B**) papillary craniopharyngioma.

MR scans of the sellar region were performed on all eligible patients via a 3.0 T scanner (Signa Excite; GE healthcare, Milwaukee, WI, USA) in our institution. Coronal contrast-enhanced T1-weighted images were used in this study, as the tumor boundaries were much clearer on the enhanced images compared to the other sequences and coronal images are commonly used in clinical work, as they can provide more information about the relationship between a tumor and the surrounding structures. The parameters of the contrast-enhanced T1-weighted imaging were as follows: time repetition = 552 ms, time echo = 10 ms, field of view = 150 mm × 150 mm, data matrix = 256 × 256, and slice thickness = 5 mm. The contrast-enhanced images were acquired within 200 s after the injection of the contrast agent, gadopentetate dimeglumine (dose: 0.1 mmol/kg). The images were exported from the picture archiving and communication system (PACS). Figure 2 shows examples of ACP and PCP, respectively.

### 2.3. Image Preprocessing and Deep Neural Network Architecture

All experiments were performed on our laboratory workstation (CPU: 2.20 GHz Intel Xeon Silver 4214; RAM: 128 Gb; GPU: 24 Gb Nvidia RTX3090; OS: Ubuntu 20.04; Intel Corporation, Santa Clara, CA, USA). The model was programmed using the Python programming language, and the codes are available at https://github.com/pytorch/vision/tree/main/torchvision/models (accessed on 19 April 2022). No modifications were made in setting the network hyperparameters and data augmentation strategy.

In preprocessing, the mean in-plane voxel sizes were resampled to 1 × 1 × 1 mm^3^ and normalized to [0, 1]. Then, the images were cropped to 50 × 50 and centered on the lesion.

**Figure 3:**
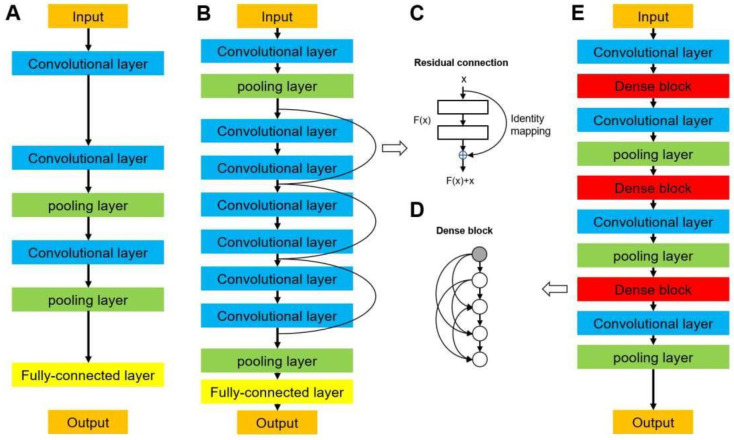
Network architectures of (**A**) a typical convolutional neural network, (**B**) a residual neural network (ResNet), and (**E**) a densely connected convolutional network (DenseNet). (**C**) Residual connection building block in ResNet. (**D**) Densely connected dense block in DenseNet.

Data augmentation was also used, where the images were randomly flipped horizontally and vertically and rotated within the range of −30 to 30 degrees for each epoch. In this study, the batch size of each architecture was set to 32. We started with a learning rate of 5E-5, multiplied it by 0.96 every six epochs, and terminated the training after 200 epochs. Three state-of-art 2D-CNN architectures were used in the current research, including VGG, ResNet, and DenseNet. They represented high-performance medical image classification, and are usually set as the ground truth in methodological research. The representative images of the CNN structure are shown in Figure 3, and a detailed description of the networks is provided below.

#### 2.3.1. VGG Networks

Visual geometry group networks (VGG) are widely used in clinical image classification, such as the diagnosis of COVID-19 and colorectal cancer [29,30]. We implemented VGG16 [31] in this study, in which 16 refers to the number of layers that have weights (Figure 3A). This network is characterized by the convolutional layers of 3 × 3 filters with a stride of 1 and the max pooling layers of 2 × 2 filters with a stride of 2. The arrangement of the convolutional layers and pooling layers runs through the entire architecture. In the end, there are two fully connected layers followed by a softmax layer for output. A residual neural network (ResNet) solves the vanishing gradient problem by providing residual connections to skip one or more layers (Figure 3B) [32], which was used in previous studies to diagnose breast lesions and predict microvascular invasion in hepatocellular carcinoma [33,34]. The plain architecture of ResNet is primarily inspired by the concept of VGG, with convolutional layers that mainly have 3 × 3 filters. As shown in Figure 3C, the residual connection acts as a simple identity mapping function, and its out- put is the same as input x. The output of the residual connection, x, is added to the output of the stacked layers, F(x, Wi); thus, a building block is defined as y = F(x, Wi) + x. In this formulation, x and y are the input and output of the building block and F(x, Wi) is the residual mapping to be learned. In this study, we implemented ResNet18, ResNet50, and ResNet101, which have 18, 50, and 101 weighted layers, respectively.

#### 2.3.2. DenseNet Networks

A dense convolutional network (DenseNet) is another CNN that introduces direct connections among all the layers [35]. They are used in the classification of diseases using clinical images, such as lung tumors and Parkinson’s disease [36,37]. Each layer obtains feature maps from all preceding layers and passes its output to all subsequent layers. As shown in Figure 3D,E, the architecture of a DenseNet contains dense blocks and transition layers. Each layer in a dense block is of the same feature-map size and implements a composite function of batch normalization, a rectified linear unit, and convolution. Because the size of the feature map is unchanged in a dense block, a transition layer that can change the feature maps is used between two contiguous blocks for downsampling, which contains a batch normalization layer, a convolutional layer (filter: 1 × 1), and an average pooling layer (filter: 2 × 2) with a stride of 2. We used DenseNet121 and DenseNet169 in this study, which were composed of 121 and 169 weighted layers, respectively.

### 2.4. Deep Learning Model Training and Test

Two-dimensional MRIs were set as the input, and the output of each case was calculated by the average probability of the slices. We applied a stratified five-fold cross-validation to evaluate the performance of the six CNN architectures. Specifically, in each fold, the numbers of ACP and PCP were 10/13 and 8/9, respectively. A receiver operation characteristics (ROC) analysis was conducted to evaluate the prediction performance of the model at all classification thresholds. The area under the ROC curve (AUC), accuracy, sensitivity, and specificity were calculated for each model. The superior model was determined as the one with the highest AUC. The performance of a network was presented with the means and standard deviations (SDs) of these metrics of the five models generated during the five-fold cross-validation.

### 2.5. Radiomics Method

**Figure 4:**
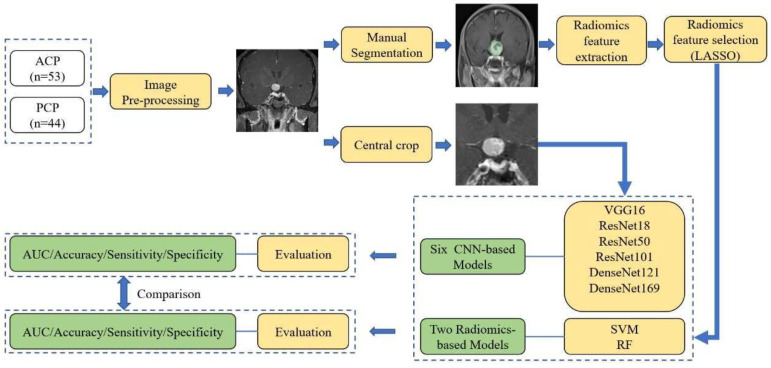
The flow chart of the current research. ACP, adamantinomatous craniopharyngioma; PCP, papillary craniopharyngioma; LASSO, least absolute shrinkage and selection operator; VGG, visual geometry group network; ResNet, residual neural network; DenseNet, densely connected convolutional network; SVM, support vector machine; RF, random forest; CNN, convolutional neural network.

Under the supervision of the senior radiologist, two researchers manually contoured the tumors slice by slice using 3D Slicer software [38]. PyRadiomics v3.0.1 was used to extract radiomics features from segmentations [39]. A total of 851 radiomics features were extracted, followed by a feature selection using a least absolute shrinkage and selection operator (LASSO) regression, a commonly used method for the regression of high-dimensional data [40,41,42]. Then, two machine-learning algorithms were employed to develop classification models, including support vector machine (SVM) and random forest (RF) [43]. The overall workflow of this research is shown in Figure 4. The AUC, accuracy, sensitivity, and specificity were calculated for each model for evaluation.

### 2.6. Statistical Analysis

The characteristics of the patients and tumors were compared between the two groups using chi-square tests and t-tests for categorical and continuous variables, respectively. All tests were two-sided, and *p* < 0.05 was considered statistically significant. All statistical analyses were conducted using Stata (version 15.1, Stata Corp., College Station, TX, USA). The CNN models were programed in the Python language and operated in the NVIDIA 3090 (NVIDIA Corporation, Santa Clara, CA, USA) data center accelerator. All radiomics algorithms were performed with R v3.6.3.

## 3. Results

### 3.1. Clinical Characteristics of the Study Population

The characteristics of the patients and tumors are summarized in Table 1. A total of 97 patients diagnosed with ACP or PCP were included in this study. Among these patients, 53 patients were diagnosed with ACP and 44 patients were diagnosed with PCP. The mean ages were 49.6 years in the ACP group and 44.7 years in the PCP group (*p* = 0.187). The male/female ratios were 1.04 and 1.44 in the ACP group and PCP group, respectively (*p* = 0.422). The mean durations of symptom onset were 50.89 weeks in the ACP group and 39.73 weeks in the PCP group (*p* = 0.453). Hypothalamic involvement was observed in 11 cases in the ACP group and 17 cases in the PCP group (*p* = 0.053). Symptomatic patients accounted for 96.2% of the ACP group and 93.2% of the PCP group. Headache, visual impairment, and endocrine dysfunction were the most common symptoms in both groups, and there was no significant differences in the distribution of symptoms.

### 3.2. Radiological Features of Tumors

The radiological features of tumors are shown in Table 1. The mean maximum diameter of ACP was significantly larger than that of PCP (37.91 mm vs. 30.02 mm, *p* = 0.015). There were no significant differences between the two groups regarding the location (*p* = 0.396), tissue component (*p* = 0.154), or shape (*p* = 0.751) of the tumors. As for the tumor location, in the ACP group, 29 patients had a tumor located in the suprasellar region, and 24 patients had a tumor in the suprasellar region with a sellar extension. In the PCP group, 27 tumors were located in the suprasellar region, 16 were located in the suprasellar region with a sellar extension, and 1 was located in the intrasellar region without suprasellar involvement. For the tumor components, 7, 15, and 31 ACPs were solid, cystic, and mixed, respectively, compared with 10, 6, and 28 of the PCPs. Moreover, 20.8% of ACPs had a regular shape, and 18.2% of PCPs were regular.

### 3.3. Prediction Performance of CNNs

**Figure 5:**
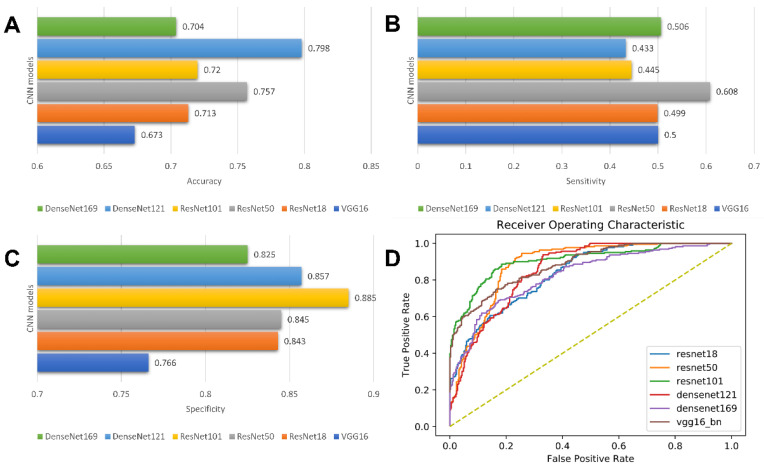
Model performance of CNN models. Bar charts of accuracy (**A**), sensitivity (**B**), and specificity (**C**); Receiver operating characteristic (ROC) curves of CNN models (**D**).

In this study, six state-of-the-art CNN architectures, including VGG16, ResNet18, ResNet50, ResNet101, DenseNet121, and DenseNet169, with a five-fold cross-validation, were implemented. Among the six deep learning models, the VGG16-based model and ResNet50-based model showed the best predictive performance, with AUC values of 0.822 and 0.838, respectively. For the model built with VGG16, accuracy = 0.673 ± 0.013; sensitivity = 0.500 ± 0.327; and specificity = 0.766 ± 0.189, respectively. For the model based on Resnet50, the accuracy, sensitivity, and specificity were 0.757± 0.052, 0.608± 0.198, and 0.845 ± 0.034, respectively. Detailed results of the model performance are summarized in Table 2 and illustrated in Figure 5.

### 3.4. Predictive Performance of Radiomics Model

**Figure 6:**
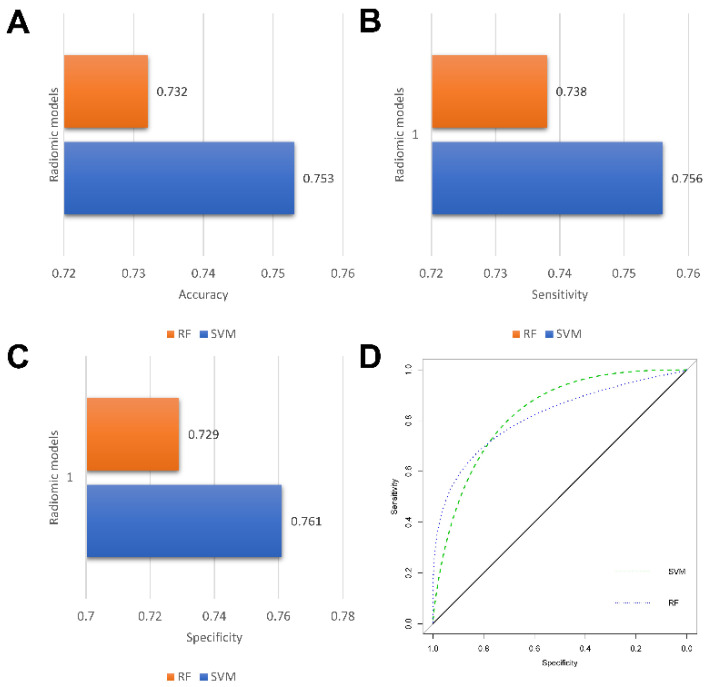
Performance of radiomics models. Bar charts of accuracy (**A**), sensitivity (**B**), and specificity (**C**); Receiver operating characteristic (ROC) curves of radiomics models (**D**).

In this study, a total of nine radiomics features were selected using a LASSO regression. The detailed radiomics features selected for each fold are listed in Supplementary Materials S1. Three machine learning models were constructed based on the selected radiomics features, with an AUC of more than 0.760 in the validation set. In a comparison of the two radiomics models, the RF-based model showed the better predictive performance, achieving the highest AUC of 0.769 ± 0.066 in the validation, while the accuracy, sensitivity, and specificity were 0.732 ± 0.053, 0.738 ± 0.044, and 0.729 ± 0.085, respectively. The detailed performances of the three radiomics models are summarized in Table 2 and illustrated in Figure 6.

In general, the CNN models performed better than the radiomics models, and the ResNet50-based model represented the best performance for the classification of ACP and PCP.

## 4. Discussion

In this study, we proposed six segmentation-free CNN models for the classification of ACP and PCP using routine contrast-enhanced T1-weighted MRIs. VGG, ResNets, and DenseNets with different weighted layers were adapted to establish CNN models. The results showed that all architectures were feasible for the discrimination of ACP and PCP and showed competitive prediction performances compared to the radiomics method. The ResNet50-based model represented the optimal architecture, with the highest AUC of 0.838, indicating that the model has the potential to help with the preoperative differentiation of ACP and PCP in adult patients and to facilitate personalized decision making for targeted therapy for craniopharyngioma.

ACP and PCP are regarded as two distinct tumors according to the 2021 WHO classification of CNS tumors [2]. First and for most, ACPs are driven by genetic mutations in CTNNB1 and have molecularly and histologically been proposed to be of embryonic origin. In contrast, PCPs harbor BRAFV600E mutations [7,44,45]. Moreover, PCP was reported to have a lower recurrence rate and mortality rate than ACP after surgical resection [46,47,48]. Conventional MRI features, such as the tumor shape, composition, location, and enhancement pattern, are also useful in the discrimination of the two subtypes of craniopharyngioma. However, previous research suggested that the machine learning model could achieve a diagnostic performance of AUC = 0.671 in validation cohorts [23]. Similarly, we found that these clinical features or MRI features in our cohort were mostly not significantly different in adult patients, suggesting that the use of MRI characteristics may be inadequate for the classification of ACP and PCP for diagnostic purposes. Previous computer-aided diagnosis (CAD) studies developed several radiomics-based machine learning models for the classification of ACP and PCP. These models were realized using selected radiomics features extracted from MRIs. One study with a total of 44 patients, by adopting a random forest classifier and four selected features, achieved an AUC of 0.89 for the classification [22]. Another multicenter study using multi-parametric MRI included a total of 164 patients, with 99 in the training group, 33 in the validation group, and 32 in the independent validation group [23]. The seven most significant radiomics features were fed into a linear support vector machine classifier, and they achieved AUCs of 0.899, 0.810, and 0.920 in the training, internal validation, and external validation groups, respectively [23]. These studies suggested that a model developed with artificial intelligence algorithms could be feasible for discriminating ACP from PCP. Compared to previous studies, the highlights of our study can be summarized as follows: First, our model was a segmentation-free end-to-end approach, indicating that human segmentation, radiomics extraction, and feature selection were not necessary in this research. Second, peritumoral regions were also included as network inputs in our research, which had been demonstrated to be helpful in predicting the characteristics of ACP [42]. Third, this study was more clinically relevant, as only adult patients were involved. The age distribution of craniopharyngioma showed significance in discrimination, as most pediatric patients were diagnosed with ACP. To make our research more clinically relevant, we excluded the pediatric patients to prevent the influence of age and only involved adult patients in this research. However, compared with previous studies with AUC values of 0.899 and 0.89, the performances of our model were slightly inadequate, with an AUC of 0.838, an accuracy of 0.757, a sensitivity of 0.608, and a specificity of 0.845 [23]. This might be attributed to the differences in the inputs into the algorithms. The model with an AUC of 0.899 was built with a multisequence feature. Previous studies suggested that, compared with single-sequence feature sets, multisequence feature sets could provide more information, and showed superior results [49,50,51]. In addition, the model with an AUC of 0.89 was built using the radiomics features extracted from high-resolution T1-w images [22]. We believe that the diagnostic performance of our CNN models could be further improved by using higher-quality images and multisequence images.

Although ResNet50 achieved the highest AUC of 0.838, the sensitivity of this model was relatively low, with a value of 0.608, and the specificity was acceptable, with a value of 0.845. This result suggested that the model was inclined to choose ACP rather than PCP in the molecular prediction. Similar results were also suggested in one previous study [23]. On the contrary, although the radiomics models showed relatively low performance, with an AUC of 0.769, all evaluation indicators showed more balanced values, with a sensitivity of 0.738 and a specificity of 0.729. Therefore, the results should be interpreted more carefully, and for neuro-radiologists and neuro-oncologists, who may need the most assistance from the intelligent model, this point should be considered when using AI models for pretreatment diagnosis.

This study had several limitations. First, this was a single-center retrospective study with inevitable selection bias. External validation in datasets of geographically diverse institutions is needed in future studies to verify the generalizability of the deep neural networks. Second, the sample size of this study was relatively small. Given the rarity of craniopharyngiomas, training models with datasets from multiple institutions is a required approach to increase the training group size and to improve the performance of neural networks in future studies. Third, only contrast-enhanced T1 images were used as CNN inputs, and the values of other sequences were unclear. Given that previous studies suggested that T1-w images played an important role in the differentiation, future studies are required to investigate if the CNN models could be improved when combined with other sequences and advanced MR technology.

## 5. Conclusions

This study proposed CNN models to discriminate ACP from PCP on MRIs. The trained models showed feasibility in the discrimination and were competitive with classical radiomics models. Drug-targeted therapy is a promising approach at the forefront of the current research in the management of craniopharyngiomas. Our models developed using deep learning technology could potentially be utilized as novel tools to assist clinicians in selecting individualized treatments for patients with craniopharyngioma.

## Figures and Tables

**Table 1:** The characteristics of the patients and tumors.

Characteristics	ACP (n = 53)	PCP (n = 44)	*p* Value
**Age (y)**	49.6 ± 3.0	44.7 ± 2.2	0.187
**Sex**			0.422
Male	27	26	
Female	26	18	
**Onset duration (w)**	50.9 ± 9.9	39.7 ± 11.0	0.453
**Symptoms**			
Headache	34	32	0.367
Visual impairment	36	35	0.198
Endocrine dysfunction	35	33	0.337
None	2	3	0.500
**Hypothalamic involvement**	11	17	0.053
**Location**			0.396
Intrasellar	0	1	
Suprasellar	29	27	
Combination	24	16	
**Tissue structure**			0.154
Solid	7	10	
Cystic	15	6	
Mixed	31	28	
**Shape**			0.751
Regular	11	8	
Irregular	42	36	
**Maximum diameter (mm)**	37.91 ± 1.7 (22–78)	30.02 ± 1.7 (9–66)	0.015

ACP, adamantinomatous craniopharyngioma; PCP, papillary craniopharyngioma.

**Table 2:** The results of the validation of the six CNNs and two radiomic models for the classification of ACP and PCP.

Method	Model	AUC	Accuracy	Sensitivity	Specificity
CNN	VGG16	0.822 ± 0.054	0.673 ± 0.013	0.500 ± 0.327	0.766 ± 0.189
	ResNet18	0.791 ± 0.055	0.713 ± 0.038	0.499 ± 0.203	0.843 ± 0.077
	ResNet50	0.838 ± 0.062	0.757 ± 0.052	0.608 ± 0.198	0.845 ± 0.034
	ResNet101	0.821 ± 0.080	0.720 ± 0.079	0.445 ± 0.122	0.885 ± 0.060
	DenseNet121	0.799 ± 0.063	0.798 ± 0.051	0.433 ± 0.052	0.857 ± 0.052
	DenseNet169	0.789 ± 0.050	0.704 ± 0.049	0.506 ± 0.088	0.825 ± 0.063
Radiomics	SVM	0.763 ± 0.068	0.753 ± 0.091	0.756 ± 0.058	0.761 ± 0.144
	RF	0.769 ± 0.066	0.732 ± 0.053	0.738 ± 0.044	0.729 ± 0.085

CNN, convolutional neural network; ACP, adamantinomatous craniopharyngioma; PCP, papillary craniopharyngioma; AUC, area under curve; ResNet, residual neural network; DenseNet, densely connected convolutional network; SVM, support vector machine; RF, random forest.

## Data Availability

The radiomic data obtained for this study are available upon reasonable request to the corresponding author.

## Supplementary Materials

The following supporting information can be downloaded at: https://www.mdpi.com/article/10.3390/jcm11247481/s1, Supplementary Materials S1: Radiomic features selected using LASSO regression in training set.
