# Supplementary material for: Pathological Diagnosis of Adult Craniopharyngioma on MR Images: An Automated End-to-End Approach Based on Deep Neural Networks Requiring No Manual Segmentation
Source: J Clin Med. 2022 Dec 16;11(24):7481. doi: 10.3390/jcm11247481 (PMC9782822; doi:10.3390/jcm11247481)
Supplement: Supplementary file 1 [file jcm-11-07481-s001.zip › jcm-2094181-supplementary.pdf]

Supplementary Materials S1: Radiomic features selected using LASSO regression in training set.

Selection Method	Selected Features
LASSO	LLL_glcm_Imc2,<br>shape_Maximum2DDiameterRow,<br>LLL_ngtdm_Busyness,<br>LLH_glcm_Idn,<br>HLH_glcm_Idn,<br>HLH_gldm_LargeDependenceEmphasis,<br>LLL_glszm_GrayLevelNonUniformityNormalized,<br>LLL_gldm_DependenceVariance,<br>HHH_glrlm_RunVariance

LASSO, least absolute shrinkage and selection operator; The detailed definitions and formulas of these features are available at https://pyradiomics.readthedocs.io/en/latest/features.html (accessed on 19 April 2022). All radiomic features were standardized to eliminate different feature magnitudes by subtracting the mean and dividing by the standard deviation.
